# Emotion Regulation Mediates the Associations of Mindfulness on Symptoms of Depression and Anxiety in the General Population

**DOI:** 10.1007/s12671-017-0709-y

**Published:** 2017-03-28

**Authors:** Laura Freudenthaler, Josephine D. Turba, Ulrich S. Tran

**Affiliations:** 1 Department of Clinical and Health Psychology, Faculty of Psychology, University of Vienna, Vienna, Austria; 2 Department of Basic Psychological Research and Research Methods, Faculty of Psychology, University of Vienna, Liebiggasse 5, 1010 Vienna, Austria

**Keywords:** Mindfulness, Emotion regulation, Depressive symptoms, Anxiety, Mediation analysis

## Abstract

In the last decade, clinical research on mindfulness and its positive effects on depression and anxiety have gained increased interest. Emotion regulation mediates the effects of mindfulness on mental health in clinical samples and among meditators. The present study examined whether these associations also generalize to the general population. Multi-group structural equation models tested with a sample of 853 adults whether difficulties in emotion regulation mediated the associations between overall mindfulness in addition to the Observe facet with symptoms of depression and anxiety and whether associations were similar among men and women. Emotion regulation partially mediated the associations of overall mindfulness with symptoms of depression and anxiety; associations with Observe were fully mediated. The magnitude of associations was similar among men and women. Mindfulness exerts positive effects on mental health among the general population mostly via improving emotion regulation. The training of mindfulness and emotion regulation may thus benefit mental health not only in clinical populations but also in the general population. Venues for further research are discussed.

## Introduction

Mindfulness, originating in the traditions of Buddhism, describes a condition of consciousness defined by a neutral and intentional focus on the current moment instead of focusing on the past or the future (Brown and Ryan 2003; Kabat-Zinn 2003). Numerous studies have shown that trait mindfulness exerts beneficial effects on mental health, such as depressive symptoms and anxiety, among both non-clinical samples (e.g., Cash and Whittingham 2010) and clinical samples (e.g., Desrosiers et al. 2013a). Mindfulness-based clinical interventions, such as mindfulness-based stress reduction (MBSR), foster mindfulness through guided meditation, body, and breathing exercises (Kabat-Zinn 2009) and are highly effective in the treatment of depression and anxiety (e.g., Khoury et al. 2013; McCarney et al. 2012; Miller et al. 1995).

Even though highly effective in both clinical and non-clinical settings, the fundamental mechanisms of mindfulness are yet not fully understood (Desrosiers et al. 2013b). However, data on associations of trait mindfulness with strategies of emotion regulation among clinical samples (Desrosiers et al. 2013b) and meditators (Tran et al. 2014) show that emotion regulation could be one path through which mindfulness unfolds its positive effects on psychological well-being.

Emotion regulation refers to strategies and processes that influence or alter the experience or expression of emotions. It covers the modulation of negative as well as positive emotions (Gross and Thompson 2007). Processes of emotion regulation can be automatic or controlled and conscious or unconscious (Gross 1998). They may aim at changing current or expected emotions concerning intensity, quality, speed of elicitation, duration, and recovery in service of adapting oneself to a situation (Thompson 1994). The process model of emotion regulation further involves situation selection, situation modification, attentional deployment, cognitive change, and response modulation (Gross 1998). As a trait, emotion regulation encompasses the capacity of cognitive control over one’s emotions, based on several different abilities (Thompson 1994). One widely used measure of emotion regulation, the DERS (Gratz and Roemer 2004), conceptualizes emotion regulation as the ability of being aware and accepting of one’s emotions, to be able to control impulsive behaviors and to behave in accordance with desired goals when experiencing negative emotions, and to be able to flexibly use emotion regulation strategies that meet individual and situational demands to modulate emotional responses.

Mindfulness practice may lead to the improvement of emotion regulation (Hölzel et al. 2011), and higher trait mindfulness was frequently reported to be associated with better emotion regulation (Desrosiers et al. 2013b; Hill and Updegraff 2012; Tran et al. 2014). A number of distinct mechanisms appear to play a role here. First, reappraisal appears relevant for mindfulness (Hölzel et al. 2011). Reappraisal entails the re-interpretation of the meaning of stimuli in order to modulate one’s emotional response. Even though reappraisal may lead to experiential avoidance and may differ on a conceptual level from a mindful state where one does not need to act on each-and-every stimulus or emotion (Chambers et al. 2009), there is evidence that trait mindfulness and reappraisal promote each other reciprocally (Garland et al. 2011). A second mechanism that appears relevant is extinction (“stimulus-response-reversal”; Hölzel et al. 2011). Experiencing a wide range of emotions, even unpleasant ones, such as fear or sadness, allows for the experience that negative emotions may fade away and make way to a sense of safety and well-being. Finally, non-reactivity to inner experiences is considered a core component of mindfulness (Baer et al. 2006) that engenders also ramifications on emotion regulation. The act of focusing on the current moment (internal and external experiences) without reacting and without judging experiences as good or bad links mindfulness to exposure therapy (Hölzel et al. 2011), which is effectively used in the treatment of anxiety disorders (Chambless and Ollendick 2001).

Deficits in emotion regulation were frequently reported to be connected with clinical depression (Ehring et al. 2010; Garnefski and Kraaij 2006; Joormann and Gotlib 2010; Radkovsky et al. 2014), as was lack of fear extinction (Anand et al. 2005). Promoting emotion regulation strategies may reduce depressive symptoms among patients with major depressive disorder (Radkovsky et al. 2014).

Prior research among persons with mood and anxiety disorders seeking treatment suggested that rumination and reappraisal mediate the association between trait mindfulness and depression, whereas worry mediates the association between trait mindfulness and anxiety (Desrosiers et al. 2013b). Rumination and worry characterize closely related forms of repetitive thinking about negative outcomes; rumination has a focus on the past and present, worry has a focus on the future. In contrast, among experienced meditators, the extent of emotional clarity and of being accepting of one’s emotions and the ability to control impulsive behaviors and to have access to a wide range of emotion regulation strategies appear to mediate the associations between trait mindfulness and depression and anxiety (Tran et al. 2014).

Symptoms of depression and anxiety are also prevalent in the general population. Currently, data on the mediating role of emotion regulation on the association between trait mindfulness and depression and anxiety in the general population are lacking. It is presently unknown whether mediating effects of emotion regulation that have been reported for clinical samples and meditators also generalize to the general population. Therefore, investigations on whether emotion regulation also mediates the positive effects of trait mindfulness on depressive and anxiety symptoms in the general population are called for. Testing associations in the general population appears further relevant, as this sets a focus on primary prevention, rather than on treatment and intervention. Furthermore, studies of Desrosiers et al. (2013b) and Tran et al. (2014) utilized the DERS, but differed in their application of the instrument. Desrosiers et al. (2013b) utilized only one of its subscales, Non-acceptance of Emotional Responses. This subscale was found an important mediator among experienced meditators (Tran et al. 2014), but not among clinical patients (Desrosiers et al. 2013b). To clarify whether the full range of strategies and abilities included in the DERS has mediating effects among the general population, utilization of the full DERS (like in Tran et al. 2014) appears necessary. As there are known sex differences in depression and anxiety (e.g., Gater et al. 1998), the possible moderating role of participant sex also needs to be controlled for in analysis.

One of the most widely used measures, the Five-Facet Mindfulness Questionnaire (FFMQ; Baer et al. 2006), conceptualizes trait mindfulness as a five-facetted construct, including Observe, Describe, Nonjudging of Inner Experience, Acting with Awareness, and Nonreactivity to Inner Experience. All facets correlate positively with adaptive characteristics (e.g., openness to experience, emotional intelligence), but Observe, unlike all the other facets, fails to be negatively correlated with maladaptive characteristics (e.g., thought suppression, experiential avoidance) in non-meditating samples (Baer et al. 2006). Among non-meditators, all facets, except Observe, are also negatively associated with symptoms of depression and anxiety; associations are often highest with Awareness and Nonjudge (e.g., Baer et al. 2006; Cash and Whittingham 2010; Tran et al. 2013). In contrast, Observe shows expected negative associations with psychological symptoms and fits into an overarching single-factor model of mindfulness only among meditating samples (Baer et al. 2006, 2008; Tran et al. 2013, 2014). Thus, it remains doubtful whether Observe may be considered a part of an overarching single mindfulness construct among non-meditators. These findings give reason to analyze Observe separately from the other facets. Desrosiers et al. (2013b) combined all five mindfulness facets to obtain a single score of trait mindfulness in their study.

The present study investigated whether emotion regulation mediated the positive effects of trait mindfulness on depressive and anxiety symptoms in the general population. We utilized the full DERS (see Tran et al. 2014) and controlled for a moderating effect of participant sex in all mediation analyses. Observe was excluded from overall mindfulness, but the scores of the four other facets were combined into a single measure, which is supported by factor-analytical evidence among non-meditating samples (Baer et al. 2006). We expected that overall mindfulness was negatively associated with symptoms of depression and anxiety in the general population. We hypothesized further that emotion regulation mediated these associations. Concerning the Observe facet, we expected that its associations with symptoms of depression and anxiety differed from the respective associations of overall mindfulness and hence needed to be modeled separately in the mediation analyses. We had no expectations regarding the direction and magnitude of possible sex differences.

## Method

### Participants

Data of 853 persons (450 women; aged from 18 to 87 years, *M* = 34.6, SD = 14.8) from the general population of Germany (45%) and Austria (44%) were used in this study (the remaining 10% were mainly from other central-European countries, 1% did not indicate their nationality). Thirty-three percent of participants indicated that they had at least some experience with mediation and mindfulness. Fourteen percent indicated that they mediated regularly (once a week or more). With regard to reported symptoms of depression and anxiety, 24% of the sample reported clinically relevant levels of depression (T score ≥63), and 20% clinically relevant levels of anxiety (T score ≥ 63), drawing on the adult norms of the BSI (Franke 2000; see Measures).

### Procedure

Data were collected by a multitude of independent data collectors through personal contacts and word-of-mouth. Participation was voluntary, anonymous, and unremunerated.

### Measures

#### Five-Facet Mindfulness Questionnaire (FFMQ)

The FFMQ (Baer et al. 2006; German form: Tran et al. 2013) consists of 39 items measuring trait mindfulness on five different subscales: Observe, Describe, Nonjudging of Inner Experience, Acting with Awareness, and Nonreactivity to Inner Experience. Respondents rate themselves on a 5-point Likert scale (1 = never, 2 = rarely, 3 = sometimes, 4 = often, 5 = always). For analysis, only items of the short version of the German FFMQ (Tran et al. 2013) were used. We selected the four items per scale that have shown the best psychometric properties with regard to factorial validity, item discrimination, and reliability, but all seven items of the Nonreactivity subscale to maximize its otherwise only low reliability (Tran et al. 2013). Items of all subscales, except Observe, were used to compute a total mindfulness score, Cronbach *α* = .85. Observe scores were computed separately, Cronbach *α* = .73.

#### Difficulties in Emotion Regulation Scale (DERS)

The DERS (Gratz and Roemer 2004) contains 36 items and six subscales that are summed to form a total score: Non-acceptance of Emotional Responses, Difficulties Engaging in Goal-directed Behavior, Impulse Control Difficulties, Lack of Emotional Awareness, Limited access to Emotional Regulation Strategies, and Lack of Emotional Clarity. Items are rated on 5-point Likert scales (1 = never, 2 = rarely, 3 = sometimes, 4 = often, 5 = always). High scores indicate difficulties in emotion regulation, low scores a relative lack thereof. Cronbach *α* of the total score was .93.

#### Brief-Symptom-Inventory-18 (BSI-18)

The BSI-18 (German form: Spitzer et al. 2011) is a short form of the Brief-Symptom-Inventory (BSI; Franke 2000). The scale assesses 18 psychological symptoms within the past 7 days on a 5-point Likert scale (0 = not at all to 4 = extremely). Three subscale scores (somatization, depression, and anxiety; six items each) and a total score may be computed. Only depression and anxiety scores were used in the present study. Cronbach *α* was .86 (depression) and .76 (anxiety).

### Data Analyses

Multi-group structural equation models were fitted to the data, testing whether DERS scores mediated the associations between overall mindfulness (FFMQ total scores without the Observe facet) and of the Observe facet with symptoms of depression and anxiety and whether associations were similar among men and women. The first model comprised both direct and indirect paths from overall mindfulness and Observe to depression and anxiety and constrained unstandardized parameter estimates to equality between men and women; depression and anxiety were allowed to correlate. In the second model, only significant paths were retained, testing specifically whether mediation was partial or full. Mplus 6.11 was used for analysis, using robust maximum likelihood estimation (MLR). For evaluation of model fit, CFI and TLI values >.95, and RMSEA values <.06, were considered to indicate a good fit.

## Results

**Fig. 1:**
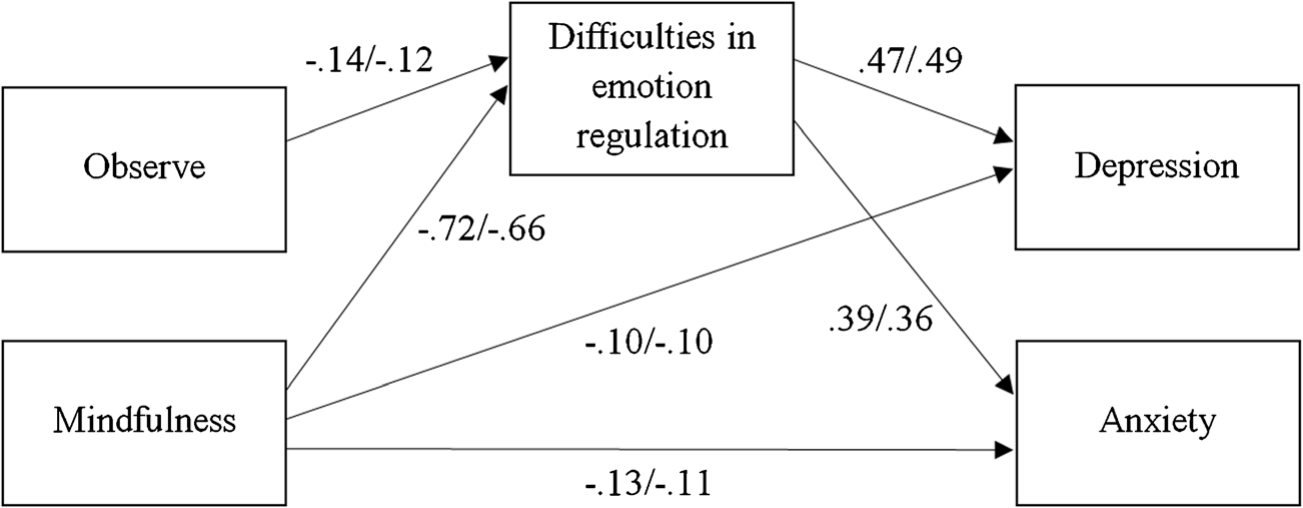
Difficulties in emotion regulation mediating the associations of overall mindfulness (FFMQ total scores without the Observe facet) and of Observe with depression and anxiety. *Numbers* represent standardized path coefficients (left/right: men/women), all *p*s < .001, except for the direct paths of mindfulness to depression (*p* = .041 and .042) and anxiety (*p* = .017 and .018). Depression and anxiety were allowed to correlate, *r* = .53 and .46, *p*s < .001. Unstandardized path coefficients were constrained to equality between men and women; standardized coefficients may still differ between groups due to differences in dispersion

Descriptive statistics are presented in Table 1. As can be seen, Observe, unlike overall mindfulness, did not correlate with measures of depression and anxiety. Its association with DERS scores was also smaller than for overall mindfulness. The first model had a good data fit (χ^2^(9) = 12.06, *p* = .212, CFI = .997, TLI = .994, RMSEA [90% confidence interval] = .028 [.000–.065]), which indicated also that associations were similar between men and women. Coefficients of direct paths of Observe to depression scores were small and not significant (standardized coefficients < .06, *p*s ≥ .090) and were similarly small for anxiety scores (among men .08, *p* = .027; among women .06, *p* = .030). Hence, direct paths of Observe to depression and anxiety scores were eliminated from the second and final model. The second model also had a good data fit (χ^2^(11) = 17.04, *p* = .107, CFI = .994, TLI = .991, RMSEA = .036 [.000–.067]). Standardized coefficients are displayed in Fig. 1. Effects of overall mindfulness on depression and anxiety were partially mediated by difficulties in emotions regulation, whereas effects of Observe appeared to be fully mediated. Coefficients of the direct paths of overall mindfulness to depression and anxiety were only small. In total, the mediation model explained 30% (men) and 31% (women) of the total variance of depression scores and 24 and 20% of anxiety scores.

**Table 1:** Intercorrelations and means and standard deviations of measured variables

					*M* (*SD*)	
	(2)	(3)	(4)	(5)	Men	Women
(1) Overall mindfulness (without Observe facet)	.15*	−.71*	−.44*	−.40*	3.41 (0.50)	3.34 (0.48)
(2) Observe		−.23*	−.06	−.01	3.55 (0.77)	3.80 (0.73)
(3) Difficulties in emotion regulation			.55*	.46*	77.73 (19.45)	78.21 (20.37)
(4) Depression				.62*	0.47 (0.66)	0.56 (0.73)
(5) Anxiety					0.50 (0.50)	0.67 (0.62)

Notes. *p < .001

## Discussion

The present study shows that emotion regulation mediated most of the effects of overall mindfulness on depression and anxiety in the German-speaking general population, corroborating and extending previous findings regarding the mediating role of emotion regulation (Desrosiers et al. 2013b; Tran et al. 2014) to a non-clinical and non-meditating sample. Consistent with prior research, higher trait mindfulness was associated with better emotion regulation (Desrosiers et al. 2013b; Hill and Updegraff 2012; Tran et al. 2014), which in turn was associated with lower depression and anxiety (see Radkovsky et al. 2014). Negative associations between overall mindfulness and symptoms of depression and anxiety were, for the biggest part, explained by emotion regulation in the present study, similarly among men and women. As remaining direct paths of overall mindfulness to depression and anxiety were only small, our results suggest that any other factor which may contribute to these associations in the general population can be only minor.

Observe differed from the other facets of mindfulness in associations with health-related outcomes and variables as expected. This confirms that the Observe facet needs to be treated separately from the other facets in the analysis of non-meditating samples (Baer et al. 2006; Tran et al. 2013) and casts some doubt on the approach to combine all five facets into an overall measure (Desrosiers et al. 2013b) for mediation analysis. Even though effects of Observe were apparently fully mediated by emotion regulation in the present study, it must be stressed that the direct effects of Observe on depression and anxiety (i.e., their bivariate zero-order correlations; see Table 1) were small and not significant. Hence, Observe contributed to lower depression and anxiety only to a negligible extent in the present sample.

Used as a preventive strategy, previous research has indicated that mindfulness-based interventions, such as MBSR and Mindfulness-Based Cognitive Therapy (MBCT; Segal et al. 2002), reduce stress and symptoms of depression and anxiety among adolescents in an educational context (Langer et al. 2015) and among employees in a workplace context (Ravalier et al. 2016). The results of the present study complement these findings and suggest that the training of mindfulness and of emotion regulation skills may be also beneficial for the prevention of symptoms of depression and anxiety in the general population. More intervention-based research in a preventive context is needed in the future.

There is evidence that different emotion regulation strategies exert significant effects on symptoms of depression and anxiety, and that mindfulness is related to depressiveness via specific facets and subscales. Acting with Awareness and Nonjudging of Inner Experience often showed the highest associations with depression and anxiety in prior research (e.g., Baer et al. 2006; Cash and Whittingham 2010; Tran et al. 2013), and some emotion regulation strategies (e.g., reappraisal, rumination, worry; Desrosiers et al. 2013b) and aspects of emotion regulation (e.g., the ability to control impulsive behaviors and to have access to a wide range of emotion regulation strategies; Tran et al. 2014) appear to be more relevant than others. Studies by Cash and Whittingham (2010) and Christopher et al. (2012) suggested that Acting with Awareness may be specifically important for depression. The present study investigated associations on an aggregate level to gain a first overall impression. Explorations on a facet level of mindfulness are needed in the future. Similarly, emotion regulation is a heterogeneous construct. In the current study, as in previous studies, emotion regulation was operationalized with the DERS, which has adequate construct validity (Gratz and Roemer 2004), but assesses specific facets and aspects of emotion regulation. Differences in the assessment and operationalization of emotion regulation need to be considered with regard to comparisons of present results with those of previous studies and need to be addressed in future studies.

## Limitations

As a general population sample, the sample of the present study included also some meditators. However, the proportion of meditators was only small and specific effects of meditation experience did not lie in the focus of the present study. Therefore, effects of meditation experience could not be differentiated, or investigated in addition to, effects of trait mindfulness. This may have affected obtained results.

Specific mechanisms of action by which emotion regulation mediated the association between mindfulness and depression and anxiety symptoms in the general population did not lie in the focus of the present study. Similarly, we did not intend to investigate effects of mindfulness facets separately, except for Observe. More research on a facet and subscale level of trait mindfulness and emotion regulation is needed in the future.
